# Dense-RefineDet for Traffic Sign Detection and Classification

**DOI:** 10.3390/s20226570

**Published:** 2020-11-17

**Authors:** Chang Sun, Yibo Ai, Sheng Wang, Weidong Zhang

**Affiliations:** 1 National Center for Materials Service Safety, University of Science and Technology Beijing, Beijing 100083, China; b20160401@xs.ustb.edu.cn (C.S.); ybai@ustb.edu.cn (Y.A.); 2 AI Lab, UCAR, 118 East Zhongguancun Road, Haidian District, Beijing 100098, China; sheng.wang03@ucarinc.com

**Keywords:** deep learning, neural network, object detection, traffic sign recognition, dense connection, anchor design

## Abstract

Detecting and classifying real-life small traffic signs from large input images is difficult due to their occupying fewer pixels relative to larger targets. To address this challenge, we proposed a deep-learning-based model (Dense-RefineDet) that applies a single-shot, object-detection framework (RefineDet) to maintain a suitable accuracy–speed trade-off. We constructed a dense connection-related transfer-connection block to combine high-level feature layers with low-level feature layers to optimize the use of the higher layers to obtain additional contextual information. Additionally, we presented an anchor-design method to provide suitable anchors for detecting small traffic signs. Experiments using the Tsinghua-Tencent 100K dataset demonstrated that Dense-RefineDet achieved competitive accuracy at high-speed detection (0.13 s/frame) of small-, medium-, and large-scale traffic signs (recall: 84.3%, 95.2%, and 92.6%; precision: 83.9%, 95.6%, and 94.0%). Moreover, experiments using the Caltech pedestrian dataset indicated that the miss rate of Dense-RefineDet was 54.03% (pedestrian height > 20 pixels), which outperformed other state-of-the-art methods.

## 1. Introduction

Traffic sign recognition plays a key role in advanced driver-assistance systems and automatic driving and is a hot topic in computer vision research and applications. Traffic sign-recognition systems are usually divided into detection and classification subtasks because signs can be first classified into different categories based on their function (such as warnings or prohibitory signs), followed by those with the same functional meaning being further classified into different subclasses according to their details [1]. Traffic sign detection aims to identify all functional categories for the signs in original images, which separates it from common object detection, which involves locating and classifying target objects simultaneously. Traffic sign classification aims to divide detected traffic signs into sub-classes [2].

The German traffic sign database is widely used for researching traffic sign detection and classification, and includes German Traffic Sign Recognition Benchmark (GTSRB) [3] and German Traffic Sign Detection Benchmark (GTSDB) [4]. However, data in the GTSRB and GTSDB do not represent real-world driving situations, because the signs in the GTSRB occupy a large proportion of the image, whereas real-world traffic signs usually occupy a smaller image area, whereas GTSDB signs only involve four categories [1]. The Tsinghua-Tencent 100K dataset is a recently proposed traffic sign benchmark that is more realistic, given that the obtained images are from vehicles and shoulder-mounted equipment [1] and has been applied in recognition methods [5,6] to evaluate their effectiveness at detecting and classifying small traffic signs.

Deep-learning-based methods have been applied for traffic sign detection and classification due to their effectiveness in feature representation [7,8,9]. Previous studies applied the convolutional neural network (CNN) either for detection or classification processes [2,9,10,11], whereas others regarded traffic sign recognition as a common object-detection task [5,6,7,12]. These methods used one CNN structure to effectively locate and classify traffic signs simultaneously; however, the challenge lies in accurately locating and classifying small traffic signs from large input images. For CNN-based methods, small traffic signs usually occupy less pixels compared with large objects, resulting in limited information contained in CNN features. Typical ways to enhance the detection of small objects include enlarging the small regions or exploiting contextual information [13]. Compared with enlarging small regions, which usually decreases speed, exploiting contextual information is preferred due to its ability to provide additional information for related target objects [14,15,16]. This method has been widely used in CNN-based small-object detection methods, such as using deconvolution or atrous convolution [17] to generate additional information [5,18,19].

A suitable trade-off between accuracy and speed is essential for traffic sign detection and classification. CNN-based object-detection methods can be classified into single-stage and two-stage methods. Two-stage methods are preferred in traffic sign recognition for their ability to achieve high detection accuracy, whereas single-stage methods achieve results with high speed. In the present study, we applied a single-stage method for traffic sign recognition and demonstrated its competitive results with state-of-the-art two-stage methods at locating and classifying real-life traffic signs. The single-shot multibox detector (SSD) [20] model is a typical single-stage method, where anchors are designed to match with objects. We found that establishing the centers of the anchors of each feature map cell as the center of the cell was not optimal for detecting small traffic signs, which motivated our use of a new anchor-design method. Additionally, to address the limited information problem, we used dense deconvolution to obtain contextual information. The resulting method based on Refinedet [21] and built on an SSD framework is named Dense-Refinedet. Our contributions are as follows:

1. We proposed an anchor-design method to detect small traffic signs using k-means clustering, followed by establishment of the center of the anchors of the shallowest feature layer at four points of each cell [namely (0.25, 0.25), (0.25, 0.75), (0.75, 0.25) and (0.75, 0.75)].
2. We built a feature-transformation module based on a dense connection in order to deliver semantic information contained in high-level layers to low-level layers and provide additional information for detecting small traffic signs.
3. Experiments using the Tsinghua-Tencent 100K and Caltech pedestrian datasets demonstrated that the Dense-Refinedet model enhanced the detection accuracy of the original RefineDet and achieved competitive performance with other state-of-the-art methods used for detecting real-world traffic signs and pedestrians.

## 2. Related Work

### 2.1. Context-Related CNN-Based Object-Detection Methods

Contextual information is important in object detection, because related objects or environment can be useful in detecting target objects [22]. Exploiting essential contextual information has been widely applied in CNN-based object detection methods. Bell et al. [15] proposed Inside-Outside Net using recurrent neural networks with rectified linear unit (ReLU) recurrent transitions and pooling multi-scale feature layers to obtain contextual information outside and inside of regions of interest (ROIs). Additionally, Zhu et al. [16] built CoupleNet by enlarging ROIs by 2-fold in order to exploit contextual information, and Li et al. [23] presented an Auto-Context R-CNN model that gained information in context-related ROIs surrounding the original region of interest (ROI).

Other methods focused on adding contextual information for small objects. Sommer et al. [24] showed that applying deconvolution to high-level features in the backbone of a Faster RCNN achieved additional context information for small objects. Cui et al. [25] described MDSSD, which forms a new fusion feature by building a skip connection between high- and low-level features (the lower feature is 4-fold smaller than the higher one). Xie et al. [19] proposed a deconvolution integrated faster R-CNN by building a deconvolution module and applying atrous convolution to generated deconvolution features in order to obtain additional contextual information for detecting small pedestrians. Lim et al. [26] described an FA-SSD model that integrated the feature-fusion technique and residual attention mechanism with a baseline SSD model by concatenating features from different layers with different scales after deconvolution and using a normalization operation to generate fusion features.

### 2.2. CNN-Based Traffic Sign-Detection and -Classification Methods

CNN-based methods for traffic sign detection and classification can be classified into those that separately or simultaneously detect and classify traffic signs. Luo et al. [9] used Maximally Stable Extremal Regions (MSERs) to extract ROIs, followed by application of a multi-tasking CNN model to refine and classify ROIs. Zhu et al. [10] proposed a traffic sign-recognition framework based on a fully convolution network (FCN) and CNN, with the FCN mainly used to extract traffic sign areas from an image, and the CNN used to classify traffic sign areas provided by the FCN. Yang et al. [2] built a real-time traffic sign-recognition system by designing a fast detection model and using a CNN to classify detected traffic signs. Habibi et al. [11] presented a lightweight and accurate traffic sign-detection method using a dilated convolution algorithm and a real-time classification ConvNet.

Other studies combined traffic sign-detection and -classification tasks into one network. Meng et al. [7] proposed an SSD-based SOS-CNN model requiring small image patches decomposed from the original image as input. Zhu et al. [1] provided a CNN model to detect and classify traffic signs simultaneously, finding this method superior to Faster RCNN at detecting traffic signs. Li et al. [8] proposed a perceptual generative adversarial network (GAN) model focused on representing small objects in a way similar to large objects by allowing its generator to obtain a super-resolved version of small objects in order to limit the difference between small and large objects. Liu et al. [6] presented a small traffic sign-detection model based on Faster RCNN (DR-CNN) by concatenating three shallow layers in a backbone via deconvolution and normalization to obtain additional information. Liu et al. [5] proposed MR-CNN, with contextual regions selected based on concatenated features in the MR-CNN to provide additional information for small objects. Noh et al. [27] proposed a GAN-based model to detect small objects by generating super-resolution features with the guidance of target features extracted from original images, after which the generated super-resolution features were used to predict small objects. Song et al. [28] presented a lightweight CNN model for mobile platforms to detect small traffic signs, with network cropping and convolutional kernel decomposition techniques applied to reduce parameters.

## 3. The Proposed Method

### 3.1. RefineDet Rrevisited

RefineDet [21] is a single-stage method based on the SSD framework and comprises an anchor-refinement module (ARM) and an object-detection module (ODM). The ARM passes negative hard-refined anchors and positive-refined anchors to the ODM, which attempts to locate and classify target objects in input images. Features in the ARM are transferred to the ODM by a designed transfer connection block (TCB), which collects two adjacent feature layers (low-level and high-level) from the ARM as input and applies a deconvolution operation to the high-level layer in order to obtain features of the same size as the low-level layer to generate fusion features by element-wise summation. The designed TCB can provide additional contextual information. In the present study, we used RefineDet as a baseline model to detect and classify traffic signs for the following reasons: (1) it is highly efficient due to its single-stage architecture; and (2) it uses a refine process that mimics a “detection process” to find possible regions in target traffic signs, regardless of their categories. Here, our “detection process” differed from that used in traditional traffic sign detection (i.e., identify all function categories specific among traffic signs in original images).

RefineDet is a powerful object-detection method used to detect objects with high speed and accuracy; however, it is not competitive with state-of-the-art methods at small-object detection. We speculate that this is due to two reasons. First, features of shallow layers in RefineDet and used to detect small objects contain limited information, which is not powerful enough to effectively detect small objects. Multi-scale features are output in RefineDet, and the shallowest feature maps with the largest resolution are scaled at 1/8 the size of the input image. Due to down-sampling convolution and pooling operations, information loss occurs, even for the shallowest output layer. Small-sized traffic signs defined in the Tsinghua-Tencent 100K dataset have a bounding-box area of <32×32 pixels; therefore, the corresponding size of the small traffic signs are <4×4 pixels, making accurate detection challenging for RefineDet. Second, anchors designed using RefineDet are not suitable for detecting small objects, because the centers of all the anchors are fixed at the center of each cell (0.5, 0.5). This would make it difficult to detect objects with a smaller size and located at the junction area of two adjacent cells or small-sized objects next to each other.

### 3.2. Framework Overview

**Figure 1:**
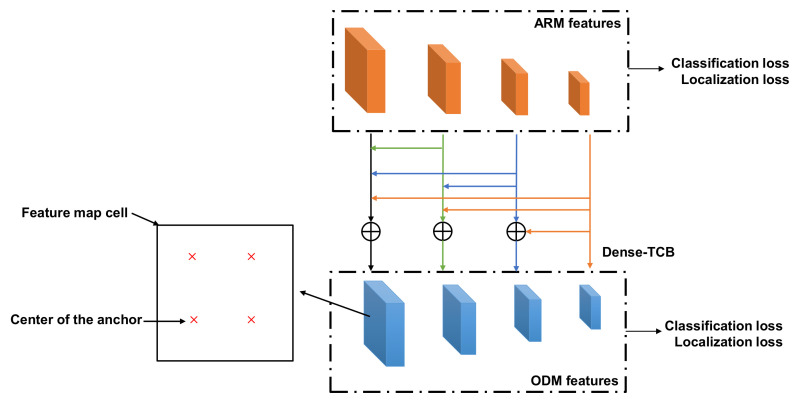
The framework of Dense-RefineDet model.TCB, transfer connection block. ARM, anchor refinement module. ODM, object-detection module.

Based on the RefineDet model, we proposed Dense-RefineDet model (Figure 1) by building a new Dense-TCB module based on a dense connection and incorporating an anchor-design method. According to RefineDet, the backbone architecture we chose was VGG-16 [29]. We output four different scale-feature maps (specifically, conv4_3, conv5_3, and conv7 layers were selected for output), and only two additional convolution layers were added, with the second extra layer used for output. The Dense-TCB module was applied to transfer additional semantic information from deeper (high-level) feature layers to shallower (low-level) feature layers and especially for the shallowest feature layer (conv4_3) in order to gain rich contextual information for detecting small-sized traffic signs. The newly designed anchors were used to address the anchor problem of RefineDet related to small-object detection.

### 3.3. Anchor Design

Anchors in RefineDet are used to locate target objects of an input image and need to be designed before the training process. For each ground-truth bounding box (GTB), the designed anchor with the highest intersection over union (IoU) value is chosen as a match. Anchors with IoU values higher than a threshold (usually 0.5) are also chosen as matches. During training, the ARM initially refines the anchors and then transfers them to the ODM. The network needs to predict the offsets between ground-truth bounding boxes (GTBs) and their matched anchors. A previous study [21] designed anchors by setting them at a constant 4-fold larger size than the stride size of each feature layer and setting three aspect ratios (i.e., 0.5, 1.0, and 2.0). Additionally, the designed anchors were placed at the center of each feature map cell. To detect small traffic signs, we found that the anchor-design method in the previous study [21] was not optimal, because the anchor shapes were suitable for objects with GTBs of different aspect ratios (i.e., objects in VOC and COCO), despite the fact that real-world traffic signs usually share similar aspect ratios. Additionally, establishing the center coordinates of the anchors at (0.5, 0.5) for each feature map cell can result in missed matches of small target objects with small-sized GTBs. In this work, the coordinates are represented as the relative coordinates of each feature map cell.

**Figure 2:**
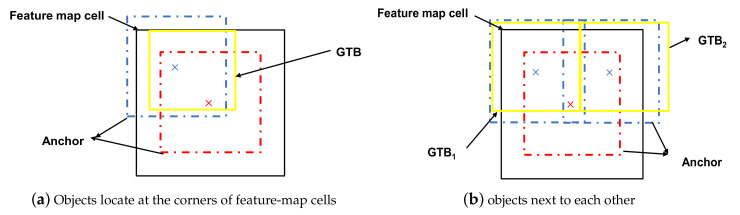
Explanation of the limitations in anchors provided by RefineDet. (**a**) Objects locate at the corners of feature-map cells, (**b**) objects next to each other.

The black boxes in Figure 2a,b are feature map cells from the shallowest feature map with the largest resolution. The yellow boxes are GTBs. The red boxes are anchors, and they have the same centers as the cells (coordinates: (0.5, 0.5)). Blue boxes share the same shape with the red anchors. In Figure 2a, the yellow GTB represents a small-sized traffic sign locates at the corner of the cell. The center coordinate of the blue anchor is (0.25, 0.75). Considering the IoU between the GTB and the blue anchor and the IoU between the same GTB and the red anchor, the blue anchor is more suitable. In Figure 2b, the yellow GTBs represent two small-sized traffic signs next to each other. The center coordinates of the blue anchors are (0.25, 0.75) and (0.75, 0.75). The red anchor can only match one of the GTBs. The blue anchors can match the two GTBs simultaneously.

To address these challenges, we proposed a new anchor-design method for detecting small traffic signs with two steps:

1. Apply k-means clustering to obtain the anchor shapes. All GTBs in the training set were used for k-means clustering to obtain anchor shapes, with k set to four.
2. Determine the anchor coordinates. Four scaled feature maps were output in our model, with the anchors shapes obtained in step 1 corresponding to different scales of the target objects. Two sets of the anchor shapes were applied to the shallowest output feature maps, and the center coordinates of these anchors were set to (0.25, 0.25), (0.25, 0.75), (0.75, 0.25), and (0.75, 0.75), making the number of anchors for the shallowest feature map cell eight. All four anchor shapes were then applied to the remaining three output feature layers with center coordinates of (0.5, 0.5), making the number of anchors for each feature map cell four.

### 3.4. Building the Dense-TCB

The TCB transfers features from the ARM to the ODM in order to provide multi-scale, fine-gained features and semantic information via deconvolution-based feature fusion. The transmission pattern in the baseline RefineDet can be expressed as Equation (1):

(1) $$O_{k}=\varphi_{k}\left(C_{k}\left(F_{l,k}\right)+D_{k}\left(F_{h,k-1}\right)\right)\;k=2,3,4$$

where the second extra layer, conv7, conv5_3 and conv4_3 layers are represented as the first (k=1), the second (k=2), the third (k=3) and the fourth (k=4) layers. $O_{k}$ is the *k*th layer of features in the ODM, $\varphi_{k}$ is a function comprising convolution and ReLU and indicating that the feature map is mapped to the ODM in a particular scale, $F_{l,k}$ is the original *k*th layer (lower-level) feature map in the ARM with larger resolution, $F_{h,k-1}$ is the original (k−1)th layer (higher-level) feature map in the ARM with smaller resolution, $C_{k}$ is the convolution related operation (including one convolution, a Relu and another convolution), and $D_{k}$ is the deconvolution operation that converts $F_{h,k-1}$ to the same resolution as $F_{l,k}$.

**Figure 3:**
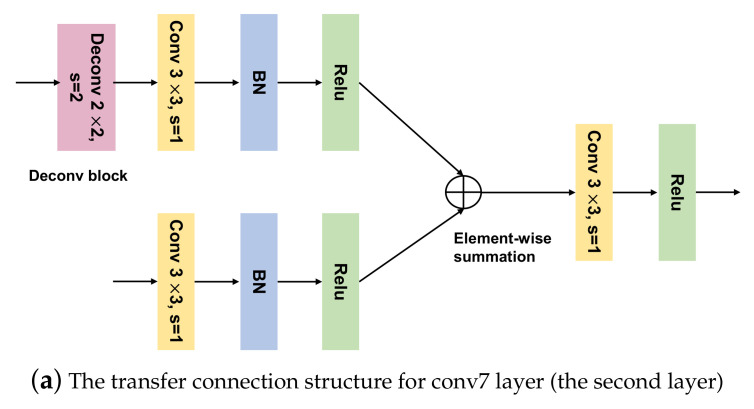

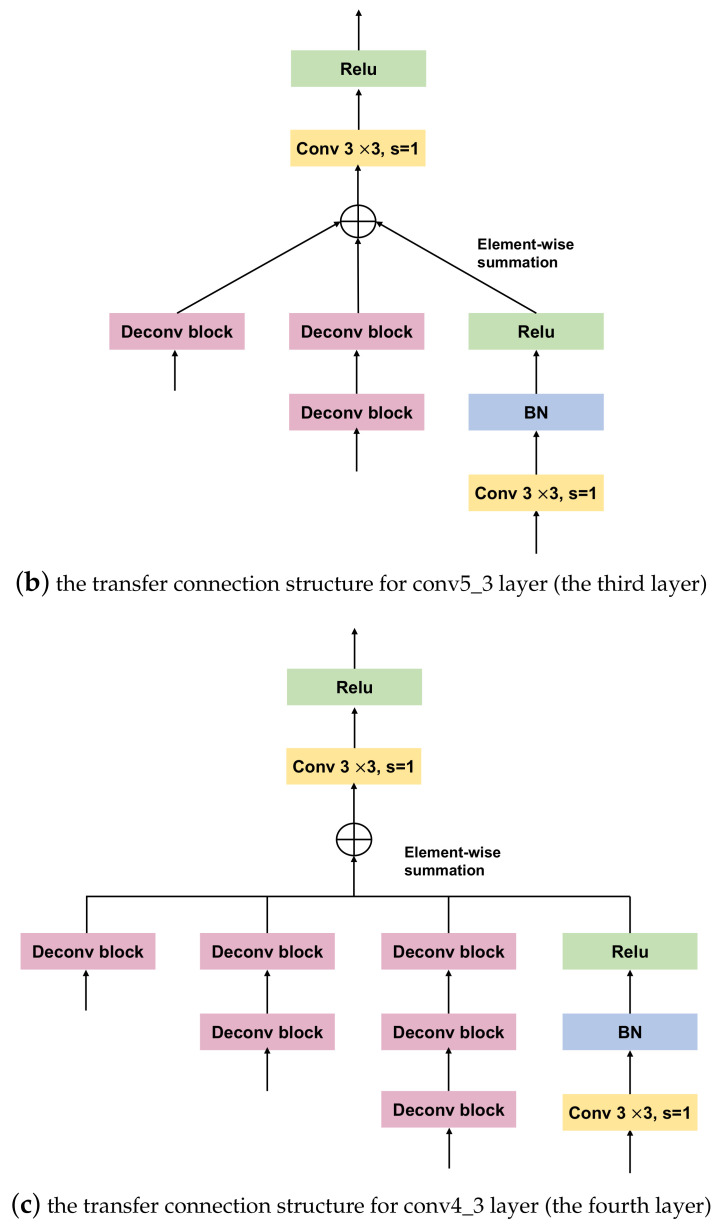
The architecture of our proposed Dense-TCB. (**a**) The transfer connection structure for conv7 layer (the second layer), (**b**) The transfer connection structure for conv5_3 layer (the third layer), (**c**) the transfer connection structure for conv4_3 layer (the fourth layer).

This transition mode of the TCB demonstrates that shallow feature maps (feature maps in lower-level layers) are gradually integrated with deep feature maps (feature maps in higher-level layers). Shallow feature maps of the CNN structure contain rich detail (spatial) information, and deep-feature maps contain rich semantic information [22,30]. Combining shallow feature maps with deep feature maps is an efficient way to obtain additional information and enhance small-object-detection accuracy [31]. To better exploit contextual information, we created a new TCB module (Dense-TCB) by embedding a dense connection [32] to improve the transmission performance of the original TCB. In Dense-TCB, the transmission principle is that all layers higher than the target layer are used to build a feature-fusion layer, which is delivered to the ODM. Compared with the original TCB, Dense-TCB can obtain more strong contextual information for detecting small objects. The proposed Dense-TCB is shown in Figure 3, and its transmission pattern is described in Equation (2):

(2) $$O_{k}={Ø}_{k}\left(B_{k}\left(F_{l,k}\right)+G_{k-1}\left(F_{h,k-1}\right)+\cdots +G_{1}\left(F_{h,1}\right)\right)\;k=2,3,4$$

where ${Ø}_{k}$ is a function that uses convolution and ReLU to output features of different scales. $B_{k}$ is the convolution related operation (including one convolution, batch normalization (BN) and a Relu), and *G* is the operation that guarantees that the features in higher-level layers have the same resolution as those in the target lower-level layer ($F_{l,k}$).

In Dense-TCB, for each feature layer in the ARM, features in the higher layers are used to generate fusion features according to a feature-fusion block (Figure 3). Four layers are output in the ARM (conv4_3, conv5_3, conv7, and the second extra layer). For the conv7 feature layer, we used one convolution (kernel size: 3×3), followed by BN and a ReLU. The deconvolution operation was applied to the second extra layer, followed by one convolution, BN and a ReLU. The two output features were then fused by element-wise summation to obtain the fusion-feature maps. One convolution (kernel size: 3×3) was applied to the fusion-feature maps to generate features transformed to the ODM. For the conv4_3 and conv5_3 feature layers, the deconvolution block in Figure 3a was replaced with blocks shown in Figure 3b,c, respectively. The extra layer was directly transferred to the ODM.

## 4. Experiments and Results

### 4.1. Datasets and Experimental Setup

Datasets: We used the Tsinghua–Tencent 100K and Caltech pedestrian [33] datasets to evaluate the performance of our proposed model. Tsinghua–Tencent 100K contains traffic signs capable of reflecting the driving situation in real life. Following the information provided by the previous study [1], we used 45 classes traffic signs. The official released training set includes 6105 images and test set includes 3071 images. The Caltech pedestrian dataset contains 11 sets of videos with the first 6 sets being training data and the rest 5 sets being test data. Following the idea in previous studies [34,35,36], 10 times augmented training images were utilized to train our model. Two versions of the annotations, the original version [33] and the new version [34], were used to evaluate our model.

Experimental setup: In this work, VGG-16 [29] was performed as backbone to build our model. The Pytorch framework was applied to train our model with an NVIDIA GeForce GTX 2080Ti GPU. We sed random cropping, distorting, expanding [20] and mirroring to augment the training images. For the Tsinghua–Tencent 100K dataset, the weight used to initialize Dense-RefineDet was trained on the ImageNet [37]. The initial learning rate was $1\times 10^{-4}$. It was decayed at 100 and 120 epochs with the decay rate $1\times 10^{-1}$. The training time was 79 h. For the Caltech pedestrian dataset, The weight used to initialize Dense-RefineDet was trained on the CityPersons dataset [38]. The initial learning rate was $1\times 10^{-4}$. It was decayed at 60 and 80 epochs with the decay rate $1\times 10^{-1}$. The training time was 16 h.

### 4.2. Detection Performance

#### 4.2.1. Performance on the Tsinghua-Tencent 100K Dataset

To evaluate the performance of Dense-RefineDet at traffic sign recognition, we compared it with other deep-learning-based methods on the Tsinghua-Tencent 100K dataset. All traffic signs were classified into three groups according to their instance sizes. Traffic signs with instance areas between 0 and $32^{2}$ pixels, $32^{2}$ and $96^{2}$ pixels, $96^{2}$ and $400^{2}$ pixels belonged to the small, medium, and the large scales, respectively. As previously described, we used precision and recall metrics to evaluate all of the methods [1,5,6,20,39,40,41,42]. As shown in Table 1, for small-scale images, the recall of Dense-RefineDet was 84.3%, which was 5.0% lower than DR-CNN [6] and MR-CNN [5] (both 89.3%), and the precision was 83.9%, which was 0.8% higher than DR-CNN and 1.0% higher than MR-CNN. For medium-scale images, Dense-RefineDet outperformed DR-CNN and MR-CNN, with recall values 0.4% and 0.8% higher and precision values 3.9% and 3.0% higher, respectively. For large-scale images, Dense-RefineDet achieved the best performance among the methods tested, with recall values 3.0% and 4.4% higher than DR-CNN and MR-CNN, respectively, and precision values 1.6% and 2.0% higher than DR-CNN and MR-CNN, respectively.

We designed the anchor shapes according to GTBs of all the traffic signs in the training set. We obtained the anchor coordinates by establishing the anchor centers of the shallowest feature layer at four points of each cell and the anchor centers of the other output feature layers at the center of each cell. Anchors were used to match with GTBs before the training process, making them full of importance. Additionally, the proposed Dense-TCB can provide output feature layers with additional contextual information by transferring information from high-level feature layers to low-level feature layers in a dense-connected way. The designed anchors and Dense-TCB enhanced the performance of our model in detecting traffic signs.

For traffic sign recognition, a suitable speed–accuracy trade-off is important. At the testing times shown in Table 1, the speed of Dense-RefineDet was 0.13 s/frame, which was competitive with that of RFB [40] (0.14 s/frame) bases on use of the single-stage detection framework. However, the detection accuracy of RFB was not as good as Dense-RefineDet, DR-CNN, or MR-CNN. The speed of Dense-RefineDet was faster than that of DR-CNN, which showed a testing time 0.13 s slower for an input image. These results showed that Dense-RefineDet was competitive with other state-of-the-art methods at detecting traffic signs.

**Figure 4:**
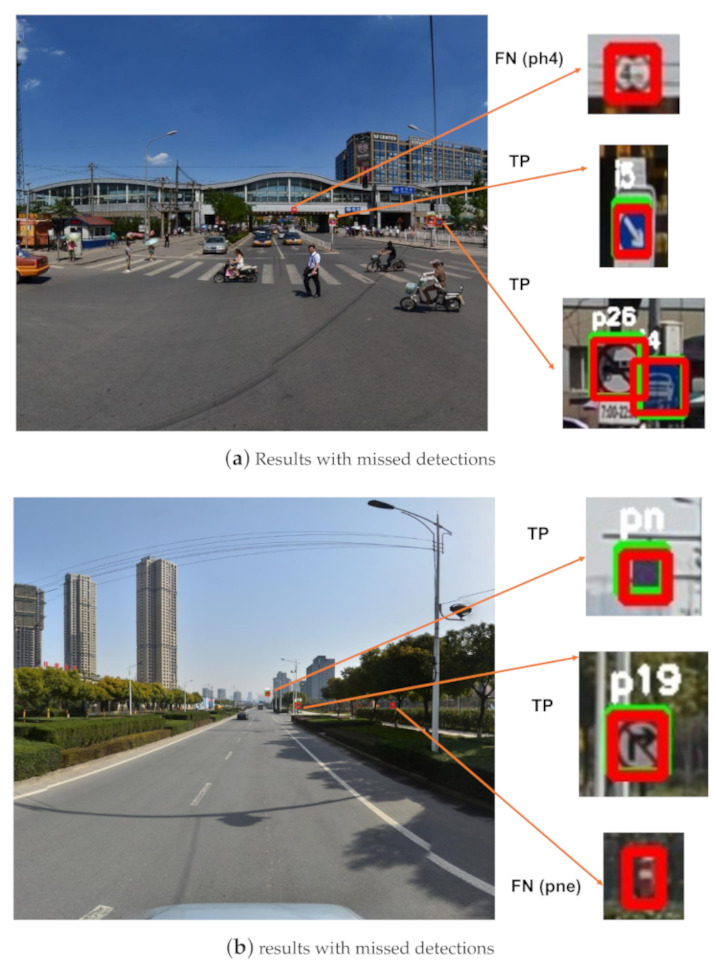

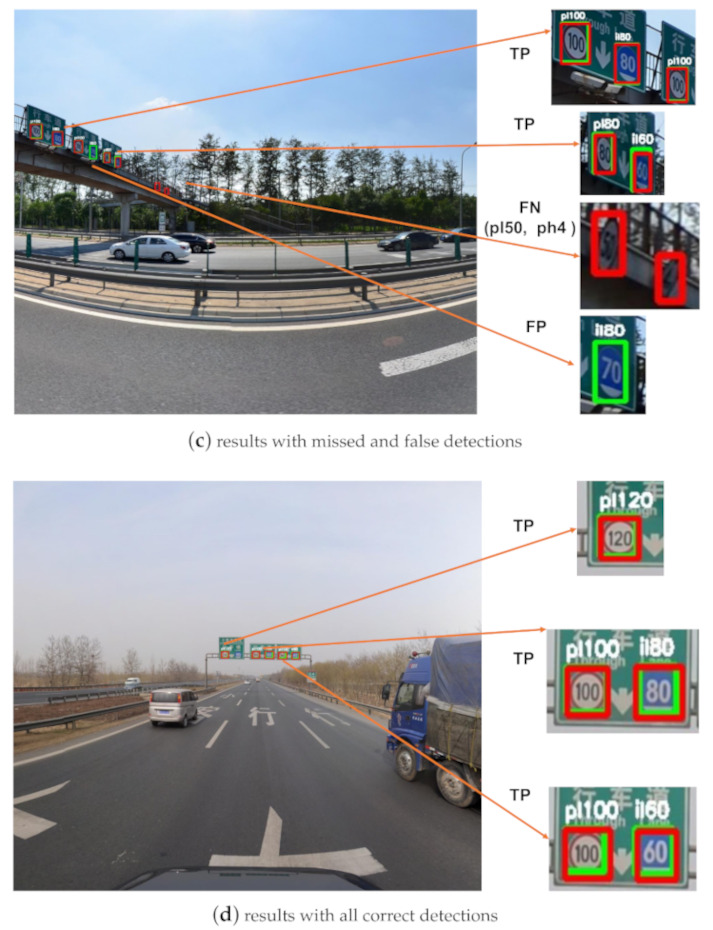
Detection results of images from the Tsinghua-Tencent 100K test set. True positive (TP), false positive (FP), false negative (FN). The red boxes are ground-truth bounding boxes, the green boxes are detection results. (**a**) Results with missed detections, (**b**) results with missed detections, (**c**) results with missed and false detections, (**d**) results with all correct detections.

Figure 4 shows some exemplary detection results for the correct and incorrect detections. We believe the cause of the false detections is that some traffic signs have a small gap between classes (the false positive detection result in Figure 4c). The main reason for the missed detections is that the pixels occupied by some small-sized traffic signs are really few, making the information used for detection is limited, such as the ’ph4’ (19×19 pixels) in Figure 4a and ’pne’ (14×22 pixels) in Figure 4b.

**Figure 5:**
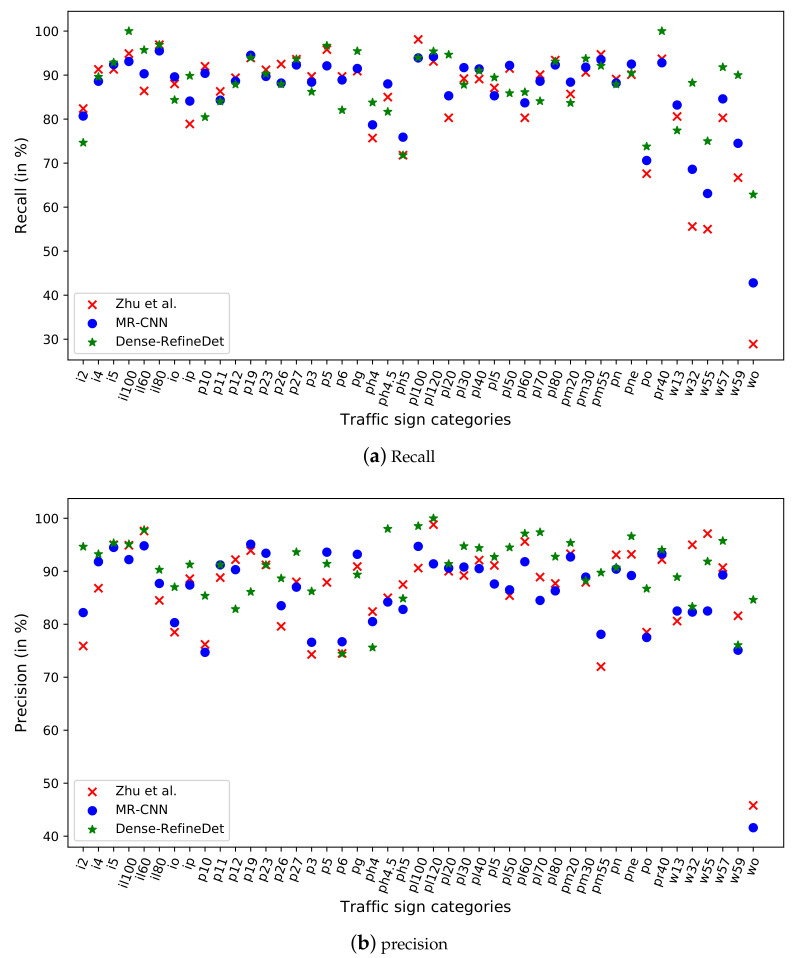
Comparisons of detection results on 45 categories. (**a**) Recall, (**b**) precision.

Furthermore, comparison with the MR-CNN model in each traffic sign category indicated that Dense-RefineDet demonstrated better recall (Figure 5a) and precision (Figure 5b), suggesting that Dense-RefineDet outperformed other methods in traffic sign detection. For traffic sign classes, such as ’p6’, ’p23’, ’p19’ and ’p12’, the recall and precision of the other two models were both higher than those of our Dense-RefineDet, or only one of the models obtained similar results with our Dense-RefineDet. We found that the instance numbers of the aforementioned traffic sign classes in the Tsinghua-Tencent 100K training set was relatively small (between 69 and 163). The detection performance of these traffic signs might be improved by new augmentation tricks.

#### 4.2.2. Performance on the Caltech Pedestrian Dataset

To demonstrate the effectiveness of our proposed Dense-RefineDet, we evaluated Dense-RefineDet with other state-of-the-art deep learning-based models [38,43,44,45,46,47,48] on the Caltech pedestrian dataset. The metric we employed was log-average miss rate (MR), which was calculated by computing average of the miss rate at false positive rates [49]. We focused on evaluating pedestrians with heights more than 20 pixels (all scale pedestrians).

**Figure 6:**
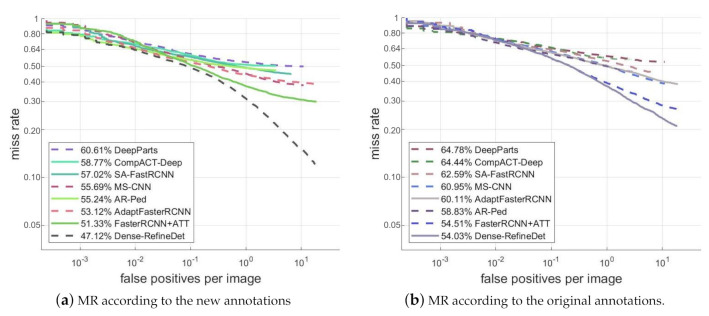
Comparisons of detection results on the Caltech pedestrian dataset. (**a**) MR according to the new annotations, (**b**) MR according to the original annotations.

For the new version annotations, we can observe from Figure 6a that the MR of Dense-RefineDet was 47.12%, which was 4.21% lower than the second-best method Faster RCNN + ATT [45]. For the original version annotations, Figure 6b indicated that the MR of Dense-RefineDet was 54.03%. It was 0.48% lower than Faster RCNN + ATT, which was 54.51%. Considering the comparison between our Dense-RefineDet and other deep learning-based models on the Caltech pedestrian dataset, we can conclude that our proposed model was capable of obtaining competitive performance.

We compared the running time of our model with other deep learning-based methods. The results are shown in Table 2. The input size of our Dense-RefineDet was 640×640. The corresponding running time was 0.06 s/frame, indicating that Dense-RefineDet was faster than other methods in detecting pedestrians.We believe the reason is that Dense-RefineDet is built on a single-stage framework.

#### 4.2.3. Ablation Study

In Dense-RefineDet, we provided a new anchor-design method and proposed a Dense-TCB module based on dense connection. To exploit the effectiveness of the new anchor-design method and the Dense-TCB module, we experimented to evaluate the associated improvements. As shown in Table 3, the newly designed anchors improved the mean average precision (mAP) by 1.30% for all traffic sign instances, and the recall and precision values were improved for small-, medium-, and large-scale traffic signs (recall: 3.25%, 5.40% and 5.91%; precision: 3.99%, 4.00%, and 3.76%). Additionally, using the armed Dense-TCB improved the mAP by 1.19% for all traffic sign instances, as well as improved the recall and precision values for small-, medium-, and large-scale traffic signs (recall: 0.35%, 1.03%, and 0.42%; precision: 0.34%, 1.22%, and 0.79%). These results demonstrated that the new anchor-design method and the Dense-TCB module effectively enhanced the detection performance of baseline RefineDet in detecting traffic signs.

## 5. Conclusions

In this study, we proposed a method for recognizing small traffic signs (Dense-RefineDet) based on RefineDet. We proposed a new anchor-design method for detecting small traffic signs located at the corners of feature-map cells or small traffic signs next to each other. A Dense-TCB was incorporated to deliver semantic information from all of the higher-level layers to the target lower-level layer and generate rich contextual information for small-sized traffic signs. Evaluation using the Tsinghua-Tencent 100K dataset demonstrated that Dense-RefineDet was competitive with state-of-the-art methods at detecting small-sized traffic signs (<$32^{2}$ pixels) and achieved better performance at detecting medium-and large-sized traffic signs ($32^{2}$ pixels < medium < $96^{2}$ pixels; $96^{2}$ pixels < large < $400^{2}$ pixels). Moreover, Dense-RefineDet was faster than other deep-learning-based methods due to its use of a single-stage framework. Furthermore, we verified the performance of Dense-RefineDet at detecting pedestrians using the Caltech pedestrian dataset, with the results confirming its competitive performance relative to other state-of-the-art methods. Our future work will improve upon the accuracy of Dense-RefineDet in detecting small-sized traffic signs and exploit its lightweight backbone for possible embedding into mobile systems.

## Figures and Tables

**Table 1:** Comparisons of detection results on the Tsinghua-Tencent 100K dataset.

Methods	Testing Time (s/Frame)	Metrics	Small	Medium	Large
Faster RCNN [39]	0.23	recall	49.8	83.7	91.2
		precision	24.1	65.6	80.8
SSD [20]	-	recall	43.4	77.5	86.9
		precision	25.3	67.8	81.5
Pon et al. [41]	-	recall	24.0	54.0	70.0
		precision	65.0	67.0	75.0
RFB [40]	0.14	recall	73.5	84.3	85.1
		precision	76.2	79.5	91.5
Zhu et al. [1]	0.77	recall	87.4	93.6	87.7
		precision	81.7	90.8	90.6
Song et al. [42]	-	recall	88.0	94.0	87.0
		precision	83.0	91.0	91.0
MR-CNN [5]	-	recall	89.3	94.4	88.2
		precision	82.9	92.6	92.0
DR-CNN [6]	0.26	recall	89.3	94.8	89.6
		precision	83.1	91.7	92.4
Dense-RefineDet	0.13	recall	84.3	95.2	92.6
		precision	83.9	95.6	94.0

The results of Faster RCNN, RFB, SSD and the model provided by Zhu et al. were published in previous studies [5,6].

**Table 2:** The computational efficiency comparison of Dense-RefineDet with other methods on the Caltech pedestrian dataset.

Methods	Input Size	MR (New)	MR (Original)	Runtime (s/Frame)
DeepParts [47]	-	60.61	64.78	1.00
SA-FastRCNN [44]	720×960	57.02	62.59	0.59
MS-CNN [43]	720×960	55.69	60.95	0.40
AR-Ped [48]	720×720	55.24	58.83	0.09
Dense-RefineDet	640×640	47.12	54.03	0.06

The run time of MS-CNN was reported in the previous study [19]. The run time of DeepParts was reported in the previous study [50].

**Table 3:** Improvements brought by the designed anchors or the proposed Dense-TCB on the Tsinghua-Tencent 1009K test set.

Metrics		RefineDet Only	RefineDet + Designed Anchors	RefineDet + Designed Anchors + Dense-TCB
mAP		80.76	82.06	83.25
Small	recall	61.38	64.63	64.98
	precision	62.72	66.71	67.05
Medium	recall	84.26	89.66	90.69
	precision	86.70	90.70	91.92
Large	recall	87.48	93.39	93.81
	precision	90.13	93.89	94.68
